# Development of a co-designed behaviour change intervention aimed at healthcare professionals recruiting to clinical trials in maternity care

**DOI:** 10.1186/s13063-022-06816-6

**Published:** 2022-10-12

**Authors:** Vivienne Hanrahan, Louisa Lawrie, Eilidh Duncan, Linda Biesty, Katie Gillies

**Affiliations:** University of Galway, Galway, Ireland

## Abstract

**Background:**

The evidence on what strategies can improve recruitment to clinical trials in maternity care is lacking. As trial recruiters, maternity healthcare professionals (MHCPs) perform behaviours (e.g. talking about trials with potential participants, distributing trial information) they may not ordinarily do outside of the trial. Most trial recruitment interventions do not provide any theoretical basis for the potential effect (on behaviour) or describe if stakeholders were involved during development. The study aim was to use behavioural theory in a co-design process to develop an intervention for MHCPs tasked with approaching all eligible potential participants and inviting them to join a maternity trial and to assess the acceptability and feasibility of such an intervention.

**Methods:**

This study applied a step-wise sequential mixed-methods approach. Key stages were informed by the Theoretical Domains Framework and Behaviour Change Techniques (BCT) Taxonomy to map the accounts of MHCPs, with regard to challenges to trial recruitment, to theoretically informed behaviour change strategies. Our recruitment intervention was co-designed during workshops with MHCPs and maternity service users. Acceptability and feasibility of our intervention was assessed using an online questionnaire based on the Theoretical Framework of Acceptability (TFA) and involved a range of trial stakeholders.

**Results:**

Two co-design workshops, with a total of nine participants (*n* = *7* MHCP, *n* = *2* maternity service users), discussed thirteen BCTs as potential solutions. Ten BCTs, broadly covering *Consequences* and *Reframing*, progressed to intervention development. Forty-five trial stakeholders (clinical midwives, research midwives/nurses, doctors, allied health professionals and trial team members) participated in the online TFA questionnaire. The intervention was perceived effective, coherent, and not burdensome to engage with. Core areas for future refinement included *Anticipated opportunity* and *Self-efficacy*.

**Conclusion:**

We developed a behaviour change recruitment intervention which is based on the accounts of MHCP trial recruiters and developed in a co-design process. Overall, the intervention was deemed acceptable. Future evaluation of the intervention will establish its effectiveness in enabling MHCPs to invite all eligible people to participate in a maternity care trial, and determine whether this translates into an increase in maternity trial recruitment rates.

**Supplementary Information:**

The online version contains supplementary material available at 10.1186/s13063-022-06816-6.

## Background

It is widely acknowledged that clinical trials frequently fail to reach recruitment targets, with just 50% of publicly funded trials in the UK achieving optimal participant numbers [1]. The consequences for trials are far reaching, including underpowered results [2], increased costs, ethical implications [3], and ultimately contribution to ‘research waste’ [4]. However, within certain clinical areas, trial recruitment can face even greater challenges, for example, in clinical trials set within maternity care [5]. The participation of pregnant people in trials differs from the general population as it potentially affects two participants. The risk of teratogenicity and adverse pregnancy outcomes increases the perception of vulnerability surrounding the mother and baby dyad [6]. Our previous work, a qualitative evidence synthesis on recruiters’ perspectives of recruiting people to pregnancy and childbirth to clinical trials, found that Maternity Healthcare Professional (MHCP) recruiters often act as protective advocates, creating an additional gatekeeping barrier between trial and participant [7]. Factors such as these have likely contributed to limiting the number of eligible people participating in maternity trials [8, 9] and the evidence available to guide researchers recruiting to clinical trials in maternity care—synergistically creating a crisis for effective recruitment for trials in maternity care.

Empirical evidence on how to recruit participants to clinical trials in general is limited, with even less evidence on which strategies are effective. In the most recent Cochrane review of strategies to improve recruitment to trials [3], the majority of strategies were targeted towards potential participants rather than Healthcare Professional (HCP) recruiters. Only three of the recruitment interventions reported were aimed at HCPs, these centred around communication [10], training and ongoing support for clinicians [11], and evaluating email or postal invitations to general practitioners [12]. In addition to a lack of HCP focus, most of the interventions in the review are atheoretical and lack a predefined mechanism of action reported during the development. The National Institute for Health Research (NIHR) recently updated framework for the development and evaluation of complex interventions (which a recruitment intervention would likely be considered) recognises the need for theory to inform intervention development [13]. As Brehaut et al., note, even large-scale methodological initiatives such as the Studies Within A Trial (SWAT) repository [14] contains studies of interventions focusing on a single aspect of recruitment that lacks a theoretical basis [15].

The trial recruitment process has multiple components [16], and many of the process components within recruitment can be considered behaviours. Behaviours such as talking about a trial with potential participants, distributing trial information, and collecting trial consent, are all behaviours that HCPs may not perform during the normal course of their role [17]. Framing trial recruitment in behavioural terms provides a structure for researchers to systematically identify which behaviours are amenable to change and target them with an intervention [18]. Using a behavioural approach to understand trial recruitment barriers has already shown promise across a variety of clinical settings [19, 20] and in trial process interventions specifically [15, 21]. While the use of behavioural theory in developing trial recruitment interventions is a relatively recent advancement [17], the theoretical grounding of this approach can offer a substantiated explanation of the barriers and solutions for trial recruitment in the maternity setting.

The aim of this study is to use behavioural theory in a co-design process to develop an intervention aimed at changing the behaviour of MHCPs recruiting to clinical trials in maternity care and to assess the acceptability and feasibility of such an intervention.

## Methods

This study is part of a larger programme of research; the ENCOUNTER project, a multi-phased, mixed methods project exploring behavioural barriers to recruitment to clinical trials in maternity care from the recruiters’ perspective and developing an intervention to target the barriers. Our previous theory-guided qualitative interview study [22] with twenty-two MHCP recruiters identified the factors enabling or inhibiting MHCP recruiters to invite all eligible women to participate in a maternity care trial. Four global themes, *Availability and accessibility of resources*, *Navigating the recruitment pathway*, *Prioritising clinical responsibilities over research responsibilities*, *and The influence of colleagues and peers*, mapped to the Theoretical Domains Framework (TDF), identified thirteen salient TDF domains relevant to the behaviour. The target behaviour of interest ‘Maternity Healthcare Professionals inviting all eligible people to participate in a maternity care trial’ was specified in our previous study [22] using the AACTT framework [23].

**Fig. 1:**
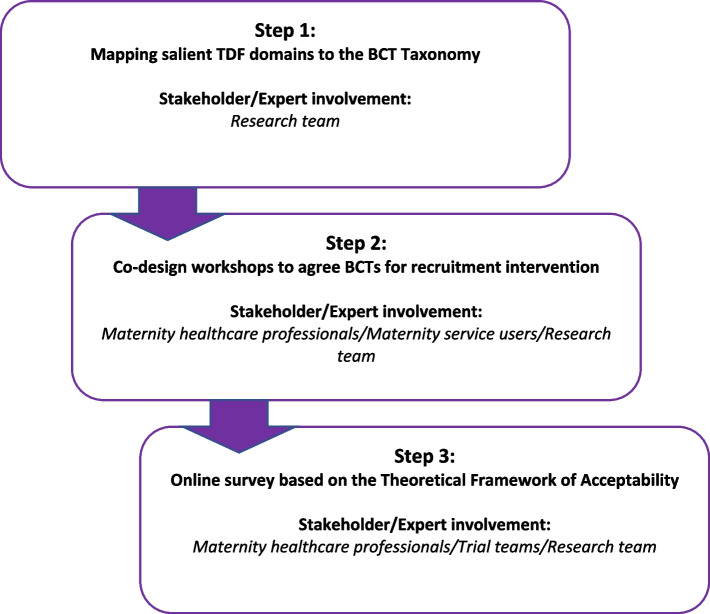
Overview of steps in intervention development

We conducted this study in three distinct steps **(**Fig. 1). Step 1 was to carry out a mapping exercise of the salient domains of the TDF to Behaviour Change Techniques (BCT) Taxonomy [24]. Step 2 was to hold a co-design workshop with stakeholders to discuss which BCTs to include in a recruitment intervention. Step 3 was to conduct an online survey to determine whether the resulting intervention was acceptable and feasible to a larger stakeholder group.

### Step 1—Mapping salient TDF domains to behaviour change techniques (BCTs)

The first step in the development of the intervention was to identify BCTs that could potentially target the salient TDF domains identified and reported in our interview study [22]. BCTs are theoretically derived and the smallest ‘active’ components of an intervention [24]. Using the online Theory and Techniques tool (an interactive ‘heat map’ matrix of 74 BCTs and 26 mechanisms of action) [25], we mapped TDF domains to BCTs, noting which BCTs were most likely to be effective in changing behaviour within a particular domain. The mapping process, undertaken by VH and LL, produced a long list of BCTs for potential use in an intervention to enable MHCPs to invite all eligible people to participate in a maternity care trial. The long list was narrowed down by the research team (VH, KG, LB) based on whether the BCT was pragmatic to operationalise within the scope of the current study (for example, non-modifiable organisational factors such as requiring additional staffing or consultation rooms were excluded). Next, suggestions were made (VH) as to how each of the BCTs shortlisted could be operationalised as a potential solution either as a stand-alone, or as part of an intervention package. For example, *BCT 7.1 ‘Prompts/cues’* could be operationalised by providing MHCPs with lanyard cards listing trial inclusion criteria. Potential solution suggestions were discussed (VH, LB, KG) and checked by behaviour change experts on the team (ED, LL) to ensure fidelity with the BCT linked to the behaviour. The APEASE (*Acceptability*, *Practicability*, *Effectiveness/cost-effectiveness*, *Affordability*, *Safety/side-effects and Equity*) criteria were applied by members of the team (VH, LB, KG) to the remaining BCTs which informed the final selection that were taken forward to the co-design workshop [26].

### Step 2—Co-design workshops for developing behaviour change recruitment intervention

In this step of the development, we included MHCP recruiters and maternity service users as stakeholders. This was to ensure the resulting intervention(s) were fit for purpose from the perspective of those who may use/receive it. We chose to hold two separate online co-design workshops, with two separate sets of participants, allowing the opportunity for each participant to contribute to the discussion.

#### Sampling

Previous participants from our interview study [22], who had consented to future contact, were invited (without obligation) to take part. We also invited maternity service users, who had experienced pregnancy within the past 2 years, via the ENCOUNTER Study Twitter account. Interested people were asked to email the research team and were sent a ‘Study Pack’ (including a brief summary of the ENCOUNTER project, workshop agenda, outline of potential solutions for discussion, and consent form). Participants returned a digital copy of signed consent. We allocated participants to one of two workshops based on their preference, clinical/professional role (midwife, nurse, doctor), the trial they were/had recruited for, and geographical location. This was to ensure, in so far as possible, that groups were balanced. Pilot workshops were held with MHCP recruiters and members of the trials community to check the coherence of the workshop content and format. Pilot participants had no previous involvement with the ENCOUNTER project, did not take part in the actual co-design workshops, nor was the data collected included in the analysis.

Workshops were held via Zoom in October 2021 and scheduled to last 90 min. Facilitation of both workshops was led by VH and co-facilitated by ED and LL. A brief summary of the ENCOUNTER project, an explanation of the ‘behavioural approach’ and workshop objectives was presented to participants using MS PowerPoint. In workshop 1, participants were asked to consider seven BCTs: *Pros and cons*, *Goal setting (behaviour)*, *Goal setting (outcome)*, *Information about antecedents*, *Self-monitoring of behaviour*, *Review behaviour goals*, and *Review outcome goals*. Each of these was presented with a description of how it could be operationalised as a potential solution to the target behaviour. Workshop 2 followed the same format, participants were asked to consider five BCTs: *Information about health consequences*, *Information about social and environmental consequences*, *Reduce negative emotions*, *Information about emotional consequences*, and *Salience of consequences.* The BCT *13.2 Reframing* was implicit in both workshops as the premise of the intervention was to conceptualise ‘recruiting’ as ‘inviting’ all eligible people to participate in a maternity trial. During both workshops, participants gave their opinion on each potential solution presented and discussed how or if they could envision the potential solution being used in practice. Finally, participants were asked if any potential solution stood out as particularly helpful or unhelpful in inviting all eligible people to participate in a maternity care trial. In using co-design principles [27], we anticipated that participants would naturally begin to discuss BCTs beyond those presented. We decided a priori to make a list of any additional BCTs suggested by participants and crosscheck them against the original long-list generated by the mapping exercise. This allowed us to reintroduce any BCTs stakeholders deemed important that had been excluded earlier.

Discussions were audio recorded to facilitate transcription and also summarised in real-time to feed back to participants at the end of the workshop (LL). Directed content analysis was performed (VH) [28], focussing on participant-reported concerns and/or advantages related to each potential solution. The summarised findings were checked by all members of the research team by comparing findings to notes taken during the workshops by LL and ED. BCTs from both workshops were brought together and VH, KG, and LB met to ensure all BCTs remained within the APEASE criteria [26]. We used the GUIDED [29] and TIDieR [30] checklists to report the development and content of the intervention (Supp. File 1).

### Step 3—Evaluating acceptability and feasibility of the proposed intervention

In the third step, we conducted an online survey to assess the acceptability and feasibility of the proposed recruitment intervention. A 3-min video introducing the proposed intervention and demonstrating a prototype of the ENCOUNTER app was recorded by the team and embedded within the online survey hosted by QuestionPro. The survey design was informed by the Theoretical Framework of Acceptability (TFA), a theoretical framework developed to assess the acceptability of healthcare interventions [31]. Participants were asked to respond using a five-point Likert scale to two belief statements and seven questions focused on each of the seven TFA constructs: *Affective attitude*, *Burden*, *Ethicality*, *Intervention coherence*, *Opportunity*, *Perceived effectiveness*, and *Self-efficacy*. An open text box was also provided for additional comments.

#### Sampling

We identified the two stakeholder groups that could potentially interact directly with the intervention: MHCPs and maternity trial team members. We invited members of these groups (based in Ireland or the UK) to complete the questionnaire. MHCPs who had taken part in or previously indicated interest in any phase of the study were emailed an invitation to participate. Chief investigators of relevant maternity care trials (identified through a clinical trials database search of Ireland and the UK) were also invited to take part. In addition, the link to the survey was promoted and shared on social media via the ENCOUNTER Study Twitter account. Consent was implicit by completion and submission of the questionnaire.

## Results

### Step 1—Mapping salient TDF domains to Behaviour Change Techniques (BCTs)

Initially, the mapping exercise resulted in 31 BCTs being identified as relevant to the target behaviour. From this long-list, 18 BCTs were excluded based on APEASE criteria. Table 1 presents further detail on the mapping process and justification for inclusion/exclusion. The remaining short-list of BCTs (*n* = 13) were then divided into two groups, broadly based on intervention function (i.e. how an intervention can change behaviour, e.g. education or modelling), to facilitate presentation to stakeholders at the co-design workshops.

**Table 1:** Results of mapping exercise (short-list of Behaviour Change Techniques (BCT) for potential inclusion in recruitment intervention)

Behavioural analysis using TDF: barriers and enablers to inviting all eligible women to participate in a clinical trial in maternity care					
**Subtheme**	**TDF domain**	**BCT (salient domain)**	**Example/suggestions**	**Intervention function(s)**	**Decision/notes**
**Availability and accessibility of resources** Having access to resources is a key enabler for trial recruitment. Recruiters need reliable technology such as iPads to facilitate mobile recruitment as they often have ‘no fixed abode’. Recruiters also struggle to communicate effectively without the use of dedicated reliable mobile phone technology. There is rarely a dedicated space for recruiters to have a private conversation with potential participants. Recruiters frequently ‘lurk’ in corridors and waiting rooms to meet potential participants. While the majority of recruiters identified the lack of space as a barrier, some felt that recruiting participants from the waiting area was an efficient use of the woman’s time. Having a sufficient number of staff, both clinical and research, allows recruiters the capacity to approach all eligible women and take the time needed to present and discuss the trial thoroughly with them. Recruiters frequently report that being understaffed limits their ability to reach all eligible women and as a result many recruitment opportunities are missed. Trial funding also plays a role in influencing recruitment efforts, as some recruiters report placing a greater emphasis on offering the trial to all eligible women, when the trial secures funding for the department	**ECR**	**3.2. Social support (practical)** *Advise on*, *arrange*, *or provide practical help (e.g. from friends*, *relatives*, *colleagues*, *‘buddies’ or staff) for performance of the behaviour*	Set up an online peer support group for recruiters Develop a trial recruiter network with regular meetings providing peer support	Enablement	Exclude*—*APEASE (effectiveness) existing practice
		**7.1. Prompts/cues** *Introduce or define environmental or social stimulus with the purpose of prompting or cueing the behaviour. The prompt or cue would normally occur at the time or place of performance*	Provide recruiters with lanyard cards listing trials and inclusion criteria	Environmental restructuring	Exclude*—*APEASE (effectiveness) existing practice
		**12.1. Restructuring the physical environment** *Change*, *or advise to change the physical environment in order to facilitate performance of the wanted behaviour or create barriers to the unwanted behaviour (other than prompts/cues*, *rewards and punishments)*	Have designated space (or folder) where trial materials are convenient and easy to access	Environmental restructuring	Exclude*—*APEASE (effectiveness) existing practice
		**12.2. Restructuring the social environment** *Change*, *or advise to change the social environment in order to facilitate performance of the wanted behaviour or create barriers to the unwanted behaviour (other than prompts/cues*, *rewards and punishments)*	Assign roles (“champions”) responsible for trial recruitment in the area. Champions would take the lead to ensuring all women were approached with trial information	Environmental restructuring	Exclude*—*APEASE (effectiveness) existing practice
		**12.5 Adding objects to the environment** *Add objects to the environment in order to facilitate performance of the behaviour*	Display trial information posters in the clinical area and staff break room (content maybe additional multiple BCTs on poster)	Environmental restructuring	Exclude*—* APEASE (effectiveness) not reaching target (AACTT^a^)
**Planning and preparation** The importance of planning and preparing for trial recruitment was emphasised. This involves screening ward and clinic lists and accessing women’s charts to gain background knowledge on potential participants. Recruiter’s favour strategies such as using a ‘two stage’ recruitment process because it allows women more time to assimilate trial information and make an informed decision as to whether they take part or not. Recruiters differentiate between non-CTIMP and CTIMP, highlighting that the latter requires extra planning and preparation on their behalf	**Behavioural regulation** Less salient domains*:* *Knowledge* *Memory*, *attention*, *decision-making processes*	**1.2. Problem solving** *Analyse*, *or prompt the person to analyse*, *factors influencing the behaviour and generate or select strategies that include overcoming barriers and/or increasing facilitators (includes ‘Relapse Prevention’ and ‘Coping Planning’)*	Provide recruiters with an opportunity to role play the recruitment encounter during trial training. Recruiters could role play with clinical colleagues to anticipate any environmental or emotional barriers and generate strategies to overcome these	Enablement	Exclude*—*APEASE (effectiveness) existing practice
		**2.3. Self-monitoring of behaviour** *Establish a method for the person to monitor and record their behaviour(s) as part of a behaviour change strategy*	Ask recruiters to keep a record of each recruitment encounter or potential encounter and the outcome*—*recruiters could then reflect on diary and share learning with peers	Enablement	Include
		**11.2. Reduce negative emotions** *Advise on ways of reducing negative emotions to facilitate performance of the behaviour (includes ‘Stress Management’)*	To overcome ‘feeling the vibe’ remind recruiters that it’s OK just to invite women to the trial by giving them the information	Enablement	Include
		**11.3. Conserving mental resources** *Advise on ways of minimising demands on mental resources to facilitate behaviour change*	Recruiters to carry with them trial information packs/lanyards cards with eligibility criteria	Enablement	Exclude*—*APEASE (effectiveness) existing practice
**Being visible** The visibility of the trial and also the recruiter is important. Recruiter’s discussed ways in which they promote the trial, using methods such as posters to raise awareness of the trial amongst clinical staff and potential participants, both internally and externally at the site. Recruiters use creative means to maintain visibility of the trial and in doing so extend their reach in accessing potential participants that might otherwise have been missed. Recruiters being visible and having a presence in the clinical area also raises awareness about the trial and gives clinical colleagues a point of contact where they can signpost potential participants to	**Behavioural regulation** **Domain was only identified as an enabler (not a barrier)** Less salient domains*:* *Intentions* *Environmental Context and resources*	**1.2. Problem solving** *Analyse*, *or prompt the person to analyse*, *factors influencing the behaviour and generate or select strategies that include overcoming barriers and/or increasing facilitators (includes ‘Relapse Prevention’ and ‘Coping Planning’)*	Encourage recruiters to plan Give recruiters time to plan and develop design recruitment strategies tailored to each trial and each site	Enablement	Exclude*—*domain was only identified as an enabler (not a barrier)
		**2.3. Self-monitoring of behaviour** *Establish a method for the person to monitor and record their behaviour(s) as part of a behaviour change strategy*	Ask recruiters to record where they placed posters etc. to monitor what works. Ask women and clinical if they have noticed these posters referring to the trial	Enablement	
		**8.2. Behaviour substitution** *Prompt substitution of the unwanted behaviour with a wanted or neutral behaviour*	Recruiters approach all eligible women about trial the rather than making an assessment beforehand Promote visibility of the trial and the recruiter by regular visits to the clinical area Recruiters hand out ‘business’ card with photo and contact details to colleagues and women	Restriction	
		**11.2. Reduce negative emotions** *Advise on ways of reducing negative emotions to facilitate performance of the behaviour (includes ‘Stress Management’)*	Reduce feelings of ‘being in the way’ by trial teams/clinical seniors reiterating the value of what they are doing and their contribution to clinical advancement	Enablement	
		**11.3. Conserving mental resources** *Advise on ways of minimising demands on mental resources to facilitate behaviour change*	Recruiters wear ID badges with trial name and recruiter role clearly visible Recruiters wear lanyards with trial name and their role clearly visible Advertise trials and brief inclusion criteria using hospital website/app	Enablement	
**Approach to recruiting** Recruiters describe how the actual approach to recruitment needs to be sensitive and appropriate to the woman’s situation. There was a common feeling that the approach should be quiet and gentle. Recruiters talk about the importance of timing when recruiting, choosing the ‘right’ time to approach was seen as key in successfully recruiting into a trial. Recruiters also discuss how their intuition plays a part in whether or not they approach a woman, describing how having a ‘feeling’ or getting a ‘vibe’ may deter them from offering a trial to a women despite her meeting eligibility criteria. Recruiters highlight that communicating the trial in a concise understandable way is key, and many describe having rehearsed a recruitment ‘spiel’ for each trial	**Emotion** Less salient domains*:* *Skills* *Beliefs about Consequences* *Environmental context and resources*	**11.2. Reduce negative emotions** *Advise on ways of reducing negative emotions to facilitate performance of the behaviour (includes ‘Stress Management’)*	Reduce feelings of apprehension (burdensome to women) by using a ‘two-stage’ recruitment strategy to introduce the trial To overcome ‘feeling the vibe’ remind recruiters that it’s OK just to invite women to the trial by giving them the information Reduce negative emotions through building peer network—discuss the balance between protecting women’s interests while supporting them to benefit from evidence-based care	Enablement	Exclude*—*APEASE (effectiveness) existing practice Include Exclude*—*APEASE (effectiveness) existing practice
		***5.5. Anticipated regret*** *Induce or raise awareness of expectations of future regret about performance of the unwanted behaviour*	Ask recruiters to assess the degree of regret they will feel if they do not invite all eligible women to participate in a trial *(ask them how they would feel if potential participants are overlooked)*	Coercion	Exclude*—*APEASE (not appropriate)
		***5.6. Information about emotional consequences*** *Provide information (e.g. written*, *verbal*, *visual) about emotional consequences of performing the behaviour*	Provide recruiters with information about women’s autonomy and right to determine their own health choices. Remind recruiters that whether to offer trial participation is not their choice to make	Education/Persuasion	Include
		***12.6. Body changes*** *Alter body structure*, *functioning or support directly to facilitate behaviour change*	Make recruiters aware of how they position themselves during a recruitment encounter. Encourage recruiters to be at the same eye level as the woman (i.e. both seated) when discussing the trial	Enablement	Exclude— APEASE (not practicable)
		***13.2. Framing/reframing*** *Suggest the deliberate adoption of a perspective or new perspective on behaviour (e.g. its purpose) in order to change cognitions or emotions about performing the behaviour (includes ‘Cognitive structuring’)*	Recruiters focus on offering the ‘invitation’ to participate in a trial (rather than thinking about the number of women that agree to participate)	Enablement	Include
**The ‘right’ participants** Despite believing that all pregnant women, if eligible, should be offered the opportunity to join a trial, and have the right to decide whether or not they participate, recruiters highlighted difficulties in identifying the ‘right’ participants. In the first instance, recruiters discuss assessing the suitability of potential trial participants based on their own judgement or beliefs on whether the woman could or would complete the trial. In the second instance, recruiters report the ambiguity relating to their own interpretation of the protocol criteria i.e. who was or was not eligible, proved problematic in finding the ‘right’ participant for the trial	**Beliefs about consequences** Less salient domains*:* *Knowledge* *Memory and decision processes* *Intentions*	**5.1. Information about health consequences** *Provide information (e.g. written*, *verbal*, *visual) about health consequences of performing the behaviour*	Explain that not inviting all eligible women to participate in a trial could restrict and slow down evidence generation and treatment options for o women during pregnancy	Education	Include
		**5.2. Salience of consequences** *Use methods specifically designed to emphasise the consequences of performing the behaviour with the aim of making them more memorable (goes beyond informing about consequences)*	Show treatment options and advances in care now available to women as a result of earlier clinical trial. Show how lack of evidence limits choices and treatment options for women	Education	Include
		**5.3. Information about social and environmental consequences** *Provide information (e.g. written*, *verbal*, *visual) about social and environmental consequences of performing the behaviour*	Remind recruiters that women have the right to be asked if they would like to participate	Persuasion	Include
		**5.5. Anticipated regret** *Induce or raise awareness of expectations of future regret about performance of the unwanted behaviour*	Ask recruiters to assess the degree of regret they will feel if they do not invite all eligible women to participate in a trial *(ask them how they would feel if potential participants are overlooked)*	Coercion	Exclude*—*APEASE (not appropriate)
		**5.6. Information about emotional consequences** *Provide information (e.g. written*, *verbal*, *visual) about emotional consequences of performing the behaviour*	Provide recruiters with information about women’s autonomy and right to determine their own health choices. Remind recruiters that whether to offer trial participation is not their choice to make	Education/Persuasion	Include
		**9.2. Pros and cons** *Advise the person to identify and compare reasons for wanting (pros) and not wanting to (cons) change the behaviour (includes ‘Decisional balance’)*	Ask recruiters to list and compare the advantages and disadvantages of inviting all eligible women to participate in a trial	Enablement	Include
		**10.10. Reward (outcome)** *Arrange for the delivery of a reward if and only if there has been effort and/or progress in achieving the behavioural outcome (includes ‘Positive reinforcement’)*	Arrange for recruiters to receive reward (i.e. box of chocs) if, and only if, there is an increase in the number of women are invited to take part in a trial	Incentivisation	Exclude*—*APEASE (ethical)
		**4.1. Instruction on how to perform behaviour** *Advise or agree on how to perform the behaviour (includes ‘Skills training’)*	Provide step by step training to recruiters on how to invite all eligible women to participate in a trial	Training	Exclude*—*APEASE (effectiveness) existing practice
		**4.2. Information about antecedents** *Provide information about antecedents (e.g. social and environmental situations and events*, *emotions*, *cognitions) that reliably predict performance of the behaviour*	Encourage recruiters to keep a record of situations or events occurring prior to approaching a woman about the trial and reflect on this (learning) with peers Experienced recruiters to give lectures/talk discussing how to troubleshoot antecedents	Education	Include
		**5.1. Information about health consequences** *Provide information (e.g. written*, *verbal*, *visual) about health consequences of performing the behaviour*	Explain that not inviting all eligible women to participate in a trial could restrict and slow down evidence generation and treatment options for o women during pregnancy. Specific tagline (easy for recruiters remember) about the health consequences of not inviting all eligible women to participate in a trial	Education	Include
		**5.3. Information about social and environmental consequences** *Provide information (e.g. written*, *verbal*, *visual) about social and environmental consequences of performing the behaviour*	Remind recruiters that not inviting all eligible women to participate in a trial could led to health inequality for women Highlight the contribution to evidence that inviting all eligible women to trial makes	Education/Persuasion	Include
**Putting women’s clinical care and wellbeing first** Prioritising the clinical care and wellbeing of potential participants is a view shared by all recruiters in the study. Trial recruitment is a secondary consideration for recruiters as they attend to the immediate physical or emotional needs that women have. Recruiters discussed their professional responsibility and a duty of care towards women and seek to minimise any potential burden associated with participation	**Intentions** Less salient domains*:* *Social/professional Role and identity* *Goals*	**Goal setting (behaviour)** *Set or agree on a goal defined in terms of the behaviour to be achieved*	Ask recruiters to set an agreed daily/weekly goal for inviting all eligible women to participate in a trial	Enablement	Include
		**5.1. Information about health consequences** *Provide information (e.g. written*, *verbal*, *visual) about health consequences of performing the behaviour*	Explain that by not inviting all eligible women to participate in a trial reduces the evidence base and treatment options available to women during pregnancy.Reinforce research as part of clinical care and central to generating evidence to inform treatments. Emphasise the counter factual is to provide a treatment based on preference not evidence, i.e. care will not be evidence based, rather it will be based on the clinicians preference	Education	Include
		**10.8. Incentive (outcome)** *Inform that a reward will be delivered if and only if there has been effort and/or progress in achieving the behavioural outcome (includes ‘Positive reinforcement’)*	Inform recruiters that they will receive money if, and only if, there is an increase in the number of women are invited to take part in a trial Acknowledge changes in behaviour (i.e. increased number of women invited to trial) by rewarding recruiters with co-authorship or other non-monetary incentives	Incentivisation	Exclude— APEASE (not acceptable)
**Acceptability of the intervention** Recruiter’s beliefs about the acceptability of the intervention directly influences whether or not they invite all eligible women to a trial. Recruiters were more comfortable and therefore more willing to recruit to trials where they believed the intervention was acceptable to women. Recruiting for trial interventions that did not align with their professional opinion was more challenging for recruiters. Furthermore, the recruiter’s perception of acceptability varies depending on their clinical background	**Beliefs about consequences** Less salient domains*:* *Social and professional role and identity*	**5.1. Information about health consequences** *Provide information (e.g. written*, *verbal*, *visual) about health consequences of performing the behaviour*	Inform recruiters of the rationale for the trial intervention and how the trial results will help fill a gap in the evidence Explain that approaching all eligible women has a potential to make trials happening more efficiently and generate evidence more quickly Address concerns about the acceptability of the trial intervention with the early involvement of recruiters (from MDT background) in design	Education	Include Exclude*—*APEASE (effectiveness) existing practice
		**5.2. Salience of consequences** *Use methods specifically designed to emphasise the consequences of performing the behaviour with the aim of making them more memorable (goes beyond informing about consequences)*	Show recruiters treatment options and advances in care now available to women because of earlier clinical trials interventions Invite past trial participants to discuss with recruiters their experiences of receiving an intervention that had been deemed ‘less acceptable’	Persuasion	Include (would need to also give balance of different opinions)
		**5.3. Information about social and environmental consequences** *Provide information (e.g. written*, *verbal*, *visual) about social and environmental consequences of performing the behaviour*	Remind recruiters that not inviting all eligible women to participate in a trial could led to health inequality for women Highlight the contribution to evidence that inviting all eligible women to trial makes	Persuasion	Include
		**5.5. Anticipated regret** *Induce or raise awareness of expectations of future regret about performance of the unwanted behaviour*	Ask recruiters to assess the degree of regret they will feel if they do not invite all eligible women to participate in a trial with an intervention that later proves beneficial to women’s care, or standard care proved to be detrimental	Coercion	Exclude*—*APEASE (not appropriate)
		**5.6. Information about emotional consequences** *Provide information (e.g. written*, *verbal*, *visual) about emotional consequences of performing the behaviour*	Recruiters share anecdotes of past emotionally sensitive situations where women were glad to be invited to participate in the trial and may have found inclusion helpful or comforting in their situation	Education/Persuasion	Include
**Commitment to the research** Recruiters express a sense of ownership towards the trial and discuss feeling ‘invested’ in it. This sense of ownership and desire for success appears to encourage recruiters to extend the invitation to participate in the trial to all eligible women Recruiters consider the research to be a worthwhile endeavour when it addresses a clinical need. Recruiters are also keen to show clinical colleagues that the research is worthwhile. Belief in the research enables recruitment, however, when doubts are raised so too are barriers	**Intentions** Less salient domains*:* *Social and professional role and identity* *Beliefs about consequences*	**1.1. Goal setting (behaviour)** *Set or agree on a goal defined in terms of the behaviour to be achieved*	Ask recruiters to set an agreed daily/weekly goal for inviting all eligible women to participate in a trial	Enablement	Include
		**5.1. Information about health consequences** *Provide information (e.g. written*, *verbal*, *visual) about health consequences of performing the behaviour*	Explain that not inviting all eligible women to participate in a trial reduces the potential evidence base and treatment options available to women during pregnancy	Education	Include
		**10.8. Incentive (outcome)** *Inform that a reward will be delivered if and only if there has been effort and/or progress in achieving the behavioural outcome (includes ‘Positive reinforcement’)*	Inform recruiters that they will receive a reward for increasing the number of eligible women invited to the trial (p*ossible reward with co-authorship/public acknowledgement*)	Incentivisation	Exclude*—*APEASE (ethical)
**Being supported** Support from peers across the trial setting is important to recruiters and enables them to offer the trial to all eligible women. Collaboration from clinical colleagues allows recruiters to gain access to potential participants, this support is valued by recruiters as they make efforts to build and nurture these relationships. Recruiters report that the absence of support from clinical colleagues passively blocks them from reaching all potential trial participants. Collaboration with other trial recruiters, both inside and outside the current trial, provides an additional source of support encouraging recruiters to offer the trial to all eligible women Recruiters reported that regular communication with the trial team was helpful, while onsite support from the team was especially appreciated	**Social Influences** Less salient domains*:* *Social and professional role and identity* *Reinforcement*	**3.1. Social support (unspecified)** *Advise on*, *arrange or provide social support (e.g. from friend*, *relatives*, *colleagues or staff or non-contingent praise or reward for performance of the behaviour. It includes encouragement and counselling*, *but only when it is directed at the behaviour*	Encourage recruiters to call a ‘buddy’ when they are struggling to invite all eligible women to the trial (i.e. set up WhatsApp group) Give recruiters protected time to engage in their peer support network	Enablement	Exclude*—*APEASE (effectiveness) existing practice
		**3.2. Social support (practical)** *Advise on*, *arrange*, *or provide practical help (e.g. from friends*, *relatives*, *colleagues*, *‘buddies’ or staff) for performance of the behaviour*	Set up an online peer support group for recruiters Develop a trial recruiter network with regular meetings providing peer support Opportunities for peer learning both within and across sites, etc Team meetings to discuss the trials (similar to MDT meeting) to get buy in from cross speciality	Enablement	Exclude*—*APEASE (effectiveness) existing practice
		**6.2. Social comparison** *Draw attention to others’ performance to allow comparison with the person’s own performance*	Show recruiters the proportion of women approached to participate in a trial by other trial recruiters/sites and compare with their own data Site league tables (publish data on all trial sites including their level of resources) at set time points in the recruitment process, so that each site can see for themselves how they compare	Persuasion	Exclude*—*APEASE (effectiveness) existing practice
		**6.3. Information about others’ approval** *Provide information about what other people think about the behaviour. The information clarifies whether others will like*, *approve or disapprove of what the person is doing or will do*	Let recruiters know clinical colleagues are on board and approve of the trial invitation	Persuasion	Exclude*—*APEASE (effectiveness) existing practice
		**10.4. Social reward** *Arrange verbal or non-verbal reward if and only if there has been effort and/or progress in performing the behaviour (includes ‘Positive reinforcement’)*	Congratulate and praise recruiters for each day/week they invite all eligible women to participate in a trial (not just for numbers recruited). Potential mode of delivery*—*Twitter/email/card to acknowledge effort	Incentivisation	Exclude*—*APEASE (effectiveness) existing practice
**Gatekeeping** Recruiter’s report experiencing some logistical and/or active clinician gatekeeping at trial sites. Much to the frustration of some recruiters, there appears to be a number of eligible women that are denied the invitation to participate in a trial because of logistical or clinician gatekeeping. While some recruiters are sympathetic and make an allowance for gatekeeping behaviour, others seek to address it by escalating the issue to senior colleagues	**Social Influences** Less salient domains*:* *Intentions*	**3.1. Social support (unspecified)** *Advise on*, *arrange or provide social support (e.g. from friends*, *relatives*, *colleagues*, *buddies or staff or non-contingent praise or reward for performance of the behaviour. It includes encouragement and counselling*, *but only when it is directed at the behaviour*	Advise recruiters to call a ‘buddy’ when they are struggling (due to gatekeeping) to invite all eligible women to the trial Arrange for trial team to support recruiters with regular site visits	Enablement	Exclude*—*APEASE (effectiveness) existing practice
		**3.2. Social support (practical)** *Advise on*, *arrange*, *or provide practical help (e.g. from friends*, *relatives*, *colleagues*, *‘buddies’ or staff for performance of the behaviour*	Set up an online peer support group for recruiters Develop a trial recruiter network with regular meetings providing peer support Encourage research culture with recruiters giving clinical colleagues regular updates on the progress of the trial and potential contribution it will make to the clinical field	Enablement	Exclude*—*APEASE (effectiveness) existing practice
		**6.2. Social comparison** *Draw attention to others’ performance to allow comparison with the person’s own performance*	Recruiters show clinical colleagues the proportion of women approached at their site compared to other sites (produce a visual representation like a thermometer chart, showing climbing numbers	Persuasion	Exclude APEASE (effectiveness) only helpful if number of women approached does in fact lead to more recruitment
		**6.3. Information about others’ approval** *Provide information about what other people think about the behaviour. The information clarifies whether others will like*, *approve or disapprove of what the person is doing or will do*	Tell recruiters that clinical colleagues approve of all eligible women being invited to participate in a trial Hold MDT trial meetings to discuss trials in the clinical area	Persuasion	Exclude*—*APEASE (effectiveness) existing practice
		**10.4. Social reward** *Arrange verbal or non-verbal reward if and only if there has been effort and/or progress in performing the behaviour (includes ‘Positive reinforcement’)*	Congratulate each clinical area for referring women to the trial (numbers could be identified by doing an audit asking women if they had been invited to participate in a trial during their care episode*—*results could be displayed in public area of hospital	Incentivisation	Exclude*—*APEASE (effectiveness) existing practice
**Recruitment targets** Recruitment targets set by the trial teams can be a driver for recruiters and encourages them to invite all eligible women to join a trial. Targets can also create competition between trial sites, which is generally well received as recruiters appreciate the opportunity to benchmark their recruitment performance. However, the element of competition serves as a disincentive for some recruiters as they feel under pressure to measure up to what they often consider as unachievable recruitment targets	**Goals** Less salient domains*:* *Reinforcement* *Emotion* *Social influences*	**1.1. Goal setting (behaviour)** *Set or agree on a goal defined in terms of the behaviour to be achieved*	Collaborative goal setting (considering resources) encouraging recruiters to set an agreed daily/weekly goal of inviting all eligible women to participate in a trial. Emphasis on SMART goals Discuss with site teams to set realistic targets	Enablement	Include (emphasis on this has to be a collaborative effort, given that unrealistic goals etc. can serve as a disincentive)
		**1.3. Goal setting (outcome)** *Set or agree on a goal defined in terms of a positive ****outcome**** of wanted behaviour*	Set a weekly goal (e.g. x number of eligible women invited to trial) as an outcome of changed recruitment behaviours	Enablement	Include
		**1.5. Review behaviour goal(s)** *Review behaviour goal(s) jointly with the person and consider modifying goal(s) or behaviour change strategy in light of achievement. This may lead to re-setting the same goal*, *a small change in that goal or setting a new goal instead of (or in addition to) the first*, *or no change*	Collaboratively review how well a recruiter’s/site’s performance corresponds to the previously agreed goals (e.g. whether they invited all eligible women to trial). Modifying future goals to ensure that they remain realistic given environmental constraints and adjust as required Review and feedback on screening logs Regular review of targets (and active support of trial teams to achieve them)	Enablement	Exclude*—*APEASE (effectiveness) existing practice
		**1.6. Discrepancy between current behaviour and goal** *Draw attention to discrepancies between a person’s current behaviour (in terms of the form*, *frequency*, *duration*, *or intensity of that behaviour) and the person’s previously set outcome goals*, *behavioural goals or action plans (goes beyond self-monitoring of behaviour)*	Trial team to do an audit and feedback on previously agreed site goals and highlight any discrepancies. The findings could be reported to recruiters and followed up with offers of practical assistance from the trial team	Coercion	Exclude— APEASE (acceptability)
		**1.7. Review outcome goal(s)** *Review outcome goal(s) jointly with the person and consider modifying goal(s) in light of achievement. This may lead to re-setting the same goal*, *a small change in that goal or setting a new goal instead of*, *or in addition to the first*	Review how many eligible women were invited to participate in a trial and consider modifying the recruitment goal accordingly, e.g. by increasing or decreasing subsequent recruitment targets	Enablement	Include

^a^*AACTT* Action Actor Context Target Time

### Step 2—Co-design workshop for developing behaviour change recruitment intervention

Twenty-two MHCP recruiters were invited to participate in one of the co-design workshop; 10 agreed to take part; due to unforeseen clinical commitments, three later withdrew. MHCPs had a range of maternity trial experience in Ireland and the UK and clinical backgrounds that included midwifery, nursing, and obstetrics. Six maternity service users expressed an interest in participating; of those, two women who had birthed within the past 6 months consented to take part. Both women had previous experience of participation in clinical research but not in a clinical trial. Further details on participant characteristics are presented in Table 2.

**Table 2:** Recruitment intervention co-design workshops: participant characteristics

Total number of participants in workshop	*n* = *9*
*Workshop 1*	*5*
*Workshop 2*	*4*
*Participants (maternity service users)*	*n* = *2*
Based in Ireland	2 (100%)
Based in UK	0
*Participants (HCP recruiters)*	*n* = *7*
Based in Ireland	1 (14%)
Based in UK	6 (86%)
*Professional background*	
Clinical midwife/nurse	0
Research midwife/nurse	6 (86%)
Obstetrician	1 (14%)
*Recruitment experience (HCP)*	
2 years	7 (100%)
< 2 years	0
*Clinical experience*	
< 5 years	1 (14%)
> 5 years	6 (86%)
*Gender*	
Female	8 (87.5%)
Male	1 (12.5%)

Workshop 1 (duration: 99 min) included five participants. Workshop 2 (duration: 106 min) included four participants. Participants offered comment on each potential solution, and the group discussed and made suggestions about how they might be adjusted, improved, or delivered in practice. Participants in both workshops suggested the intervention could be delivered as part of mandatory training. However, the majority of participants were strongly opposed to this idea, stating that the agenda for mandatory training days was already full and adding something further would necessitate losing something equally important. Participants were concerned that in the current climate (COVID-19 pandemic) MHCPs did not have the appetite for more training and it would become “just another ‘thing’ they had to do”.

Many participants commented on how mobile phone apps had recently become a useful tool in recruitment. Voice notes were discussed as a particularly helpful method of communication between MHCP recruiters. Group members shared scenarios where they regularly used voice notes to instruct other recruiter colleagues about how to communicate trial specific information to potential participants. One participant had found voice notes especially useful as a means of facilitating remote recruitment, by communicating directly with women about the trial during the COVID-19 pandemic.

Following a discussion summary, participants were asked if they considered any potential solution to be particularly helpful or unhelpful in inviting all eligible people to participate in a maternity trial. In workshop 1, the majority agreed that having ‘experienced recruiters give a talk (or video clip) about resolving situations that commonly occur prior to inviting a woman to a trial’ (*BCT 4.2—Information about antecedents*) was the most helpful. There was no agreement on the least helpful potential solution as participants all selected different BCTs. In workshop 2, participants agreed that all the potential solutions presented to them would be helpful in addressing the barriers. Furthermore, participants creatively suggested that some solutions could be combined to make a more practicable intervention. Tables 3 and 4 provides a brief summary of participant discussion around each potential solution presented at the workshops.

**Table 3:** Summary of trial recruitment intervention co-design workshop discussion with maternity healthcare professionals and maternity service users: workshop 1

Recruiter’s list and compare advantages and disadvantages of inviting all eligible women to participate in a trial **9.2 Pros and cons** *(linked subthemes—the ‘right’ participants and acceptability of the intervention)*	• Good to do at start of study, discuss how to approach and reassess once study is underway • Depends on resources and capacity to screen women to do this • Inviting all women promotes research*—*helpful for future studies • This is not something consciously done at the moment • Important to include underrepresented, non-English speaking, ethnic, and cultural minorities • Wouldn’t use this method*—*prefer team-based approach to make trial more appealing, educate public about what a trial involves • Tool is more helpful for trials difficult to recruit to or struggling*—*could modify approach by recognising problem
Set an agreed daily/weekly goal to invite all eligible women **1.1 Goal setting (behaviour)** *(linked subthemes—putting women’s clinical care and wellbeing first and commitment to the research and recruitment targets)*	• Setting targets helps*—*nice to be first to recruit and gain sense of achievement • Depends on trial, some trials you really can’t set goals for (lack of staff, not enough patients, etc.) • Tool could be helpful in identifying where problems are*—*should be reward rather than punishment system • Goals depend on staff availability and staff to support women throughout the trial ‘• Goals’ focuses on quantity not quality*—*talking to everyone spreads yourself too ‘thin on the ground’ • ‘Goals’ not good, means just another pressure*—*wouldn’t benefit the trial • Better to concentrate on areas to target (ward, clinics etc.) for recruitment rather than numbers
Set an agreed weekly goal to have increased the number of women invited to the trial **1.3 Goal setting (outcome)** *(linked subthemes—recruitment targets)*	• This one is about incentivising people to approach more women than they are doing currently • If you are recruiting well, you do not need this • Inviting everyone could be a tick box exercise (invitees may not sign up for study) • Most multicentre sites do not know how many women have been invited*—*screening logs are not routinely looked at • Tool might be useful on a local level to know if you are targeting the right areas • Tool is useful if talking to lots of women means lots of recruits • It is the quality of the chat not the numbers invited*—*its deeper than just numbers • Goals motivate people; it gets people working together on something • It would be more beneficial to look back retrospectively to learn rather than set goals
Experienced recruiters give talk (or video clip) about resolving situations that commonly occur prior to inviting a woman to a trial **4.2 Information about antecedents** *(linked subthemes—planning and preparation and being visible and benefit of experience)*	• Include people from different backgrounds, recruiters from ethnic minorities have different experiences and are asked different questions • This could cover a range of difficulties faced*—*talk through and present ways of resolving those • Also helpful in deflecting concerns and uncertainties in the clinical team*—*addressing these head on is sensible • May not video but a meeting or something at local level*—*because issues differ from site to site • Could be PI, R&D lead, research midwife/nurse delivering • Could be virtual conservation so people could ask questions, and provide recording to watch back • Zoom meetings for recruiters are held for some trials but are not always accessible as timing does not always suit staff • WhatsApp voice notes are helpful for recruiters to communicate their methods to other recruiters • Good idea to have one video at set up and one subsequently as may need to adapt approach as trial goes on
Recruiters keep diary of recruitment or potential recruitment encounters (reflecting on diary and shared learning with peers) **2.3 Self-monitoring of behaviour** *(linked subthemes—being visible and planning and preparation)*	• Writing in diary is useful, means you will remember • Write significant encounters, difficulties, or learnings*—*do not want to record every single thing • Fill them out when you have time*—*can be brief as they need to be • Diary is reflective and more useful than a screening log • Could be recorded centrally or as a personal record*—*Sharing diary is not problematic (all agreed) • Positive consequence*—*the record shows difficulties or missed opportunities which could justify argument for more resources • Tool could be helpful to show R&D why recruitment is problematic • Not sure reflection is helpful*—*if you are recruiting well why are you reflecting on it? • Diary provides retrospective perspective, helpful in identifying where women could potentially be missed
Regular review (with trial team) of the change in behaviour being used by recruiters **1.5 Review behaviour goals** *(linked subthemes—recruitment targets)*	• Might be helpful if you are not recruiting well*—*see where you could improve • Not regular review*—*better to positively motivate people with degree of healthy competition • Trial team already send league tables*—*giving shout out to top recruiter • On a local level you can incentivise with chocolates, etc.*—*small stuff means something • Already being done*—*it really helped concentrating on where is focus efforts • Review of behaviour change ensures you are doing your best and change if necessary
Trials teams carry out monthly review to reveal if all eligible women had been invited to the trial **1.7 Review outcome goals** *(linked subthemes—recruitment targets)*	• This is not achievable as there is no record of ‘all women’*—*not possible to know about all eligible women • You already know if you have approached all eligible women, and if not, you know why • This is not useful because capacity of the team is often the problem • Review helps people write down the issues and address them • A positive consequence might be reallocation of staff following review

**Table 4:** Summary of trial recruitment intervention co-design workshop discussion with maternity healthcare professionals and maternity service users: workshop 2

Inform recruiters that research is part of clinical care, explain the importance of maternity care research and give the rationale for trial **5.1 Information about health consequences** *(linked subthemes—the ‘right’ participants and benefit of experience and acceptability of the intervention and commitment to the research)*	• Research should be considered part of clinical care • COVID, through news and social media, has helped push importance of clinical research to the fore front • Work is now being done to promote research as part clinical care*—*research feels more embedded in clinical practice than before COVID*—*this needs to continue as both branches of same tree • Challenging and burdensome for research staff to educate clinical colleagues that women deserve research opportunities • Helpful to show a direct link between research and clinical care
Remind recruiters that all eligible women should be offered the chance to participate **5.3 Information about social and environmental consequences** *(linked subthemes—‘the ‘right’ participants and benefit of experience and acceptability of the intervention)*	• Really good idea*—*research invitation should be embedded like flu vaccine or whooping cough vaccine • Need to be conscious of this*—*it is hard to talk about and hard to admit • Healthcare services should prioritise translated/plain English versions of all medical information is available to women*—*so that if communication is difficult, she has something to go home with and discuss with others • Educate the research team to understand exactly why the trial is important and why it is a better option to offer it to everyone • Particularly relevant for bereaved women*—*participating might give some meaning to their experience • Mandatory training could be good delivery mode - reenforces research as part of clinical care, everybody is offered the same health advice, they should be offered research participation (i.e. all staff receive it, does not feel personal, gives people time to ask questions, not trial specific, just promoting awareness) • Mandatory training is relentless • Messages needs to come from senior staff*—*clinical leaders rather than researchers • Could have big campaign using up to date social media, i.e. Instagram and TikTok
Remind recruiters that it is OK to invite women by just giving them information **11.2 Reduce negative emotions** *(linked subthemes—planning and preparation and being visible and approach to recruiting)*	• Really helpful as there is a lot of pressure to recruit to targets*—*ongoing funding and posts demand on accruals • Pressure to recruit and limited time leads recruiters to approach only those they believe more likely to take part • Just giving information increases likelihood of recruitment • Ok if they decline*—*shows you have given them an informed choice • Good for women to receive information without feeling pressured to participate • Would not recruit successfully just by giving information*—*would need to follow up • Important to give information that is accessible to all women (languages, literacy levels, etc.) • Instagram influencers are highly regarded source of medical information for women in pregnancy*—*good medium to communicate research message through
Invite recruiters to share anecdotes of inviting women to participate in a trial during emotionally sensitive situations **5.6 Information about emotional consequences** *(linked subthemes—approach to recruiting and the right participants and acceptability of the intervention)*	• Really helpful idea • Daunting when first starting to recruit*—*speaking with person who knows how to discuss topic makes it much easier • Good to have training on how to speak to someone in this situation • This often happens informally as every situation is different*—*maybe just space and time with colleagues? • Midwifing the woman*—*she needs us, precious relationships to nurture • Audio clips (WhatsApp notes) have been useful maybe this could be developed?
Invite previous trial participants to share their experiences of being invited to a maternity care trial **5.2 Salience of consequences** *(linked subthemes—the ‘right’ participants and acceptability of the intervention)*	• Really important*—*why do not we do this more?*—*good opportunity to celebrate and share the findings • Does not necessarily need to be trial specific • Clinical midwives invite women back to share experiences in antenatal/postnatal groups • Women that have been through trials are our greatest asset in encouraging others to participate • People really crave human face-to-face or online experience*—*use Instagram? • Suggestion*—*using Teams or Zoom setting (similar to workshop) would be good • Current approach is old fashioned (giving leaflets, etc.) people do not communicate that way anymore

Six additional BCTs were mentioned by participants during the discussions, all of which featured on the initial long-list generated during the mapping exercise in step 1. Five BCTs had already been excluded based on the APEASE criteria and were not carried forward. The remaining one (*10.4 Social rewards*) was brought forward to the next stage of development.

The APEASE criteria were applied to the aggregated findings of both co-design workshops: this produced the final list of BCTs for inclusion in the intervention (Tables 5 and 6). In total, 10 BCTs were confirmed for inclusion in the final intervention (*1.2 Problem Solving*; *2.3 Self-monitoring of behaviour*; *4.2 Information about antecedents*; *5.1 Information about health consequences*; *5.3 Information about social and environmental consequences*; *5.6 Information about emotional consequence*; *9.2 Pros and Cons*; *10.4 Social Rewards*; *11.2 Reduce negative emotions*; *13.2 Reframing*).

**Table 5:** APEASE criteria applied by ENCOUNTER team to behaviour change techniques following co-design workshops: intervention 1

BCT	Label	Affordability	Practicability	Effectiveness	Acceptability	Side-effects/safety	Equity	Decision Yes/no	Reasons for exclusion/comments
9.2	**Pros and cons**								
	Description: *Ask recruiter’s list and compare advantages and disadvantages of inviting all eligible women to participate in a trial*	**√**	**√**	**-**	**√**	**√**	**√**	Yes	Perceived as helpful by most participants
1.1	**Goal setting***—***behaviour**								
	Description: *Ask recruiters to set an agreed daily/weekly goal to invite all eligible women (not necessarily the numbers)*	**√**	**X**	**-**	**X**	**-**	**√**	No	Negative response at workshop
1.3	**Goal setting***—***outcome**								
	Description: *Ask recruiters to set weekly goal to have increased the number of women invited to the trial*	**√**	**X**	**-**	**X**	**-**	**√**	No	Negative response at workshop
4.2	**Information about antecedents**								
	Description: *Show recruiters a video clip of an experienced recruiter—what prompts/motivates them to invite all eligible women to a trial as well as discussing the common challenges in approaching potential participants*	**√**	**√**	**-**	**√**	**√**	**√**	Yes	The most well received BCT at the workshop
2.3	**Self-monitoring of behaviour**								
	Description: *Ask recruiters to keep a diary of each recruitment encounter or potential encounter and the outcome (perhaps limited to the first 10). Recruiters could later reflect on diary and share learnings with peers*	**√**	**√**	**-**	**√**	**√**	**√**	Yes	Well received by midwives and nurse in the workshop
1.2	**Problem solving**								
	Description: *Encourage recruiters to be creative in thinking about strategies to adopt as challenges are presented i.e. “If x goes wrong*, *then this is what you do”*	**√**	**√**	**-**	**√**	**√**	**√**	Yes	Could fit neatly with 2.3 and the shared learnings
1.5	**Review behaviour goals**								
	Description: *Regular review*, *with the trial team*, *of the changed behaviour being used by the recruiters*	**√**	**√**	**-**	**X**	**X**	**√**	No	No longer relevant as 1.1 and 1.3 have been excluded
1.7	**Review outcome goals**								
	Description: *Trial teams carry out monthly review to reveal if all eligible women had been invited to participate in the trial. Based on outcome*, *recruiter and trial team could consider modifying invitation goal*	**√**	**X**	**-**	**X**	**X**	**√**	No	No longer relevant as 1.1 and 1.3 have been excluded
10.4	**Social rewards**^a^								
	Identified as ‘creep’ BCT Description: *Trial team give recruiters thank you cards/acknowledgements*	**√**	**√**	**√**	**√**	**√**	**√**	Yes	

^a^Additional ‘creep’ BCTs that emerged during the workshop, they were identified on original ‘Long List’ but not initially proposed as intervention components during the workshop

**Table 6:** APEASE criteria applied by ENCOUNTER team to behaviour change techniques following co-design workshops: intervention: intervention 2

BCT	Label	Affordability	Practicability	Effectiveness	Acceptability	Side-effects/safety	Equity	Decision Yes/no	Reasons for exclusion/comments
5.1	**Information about health consequences**								
	Description: *Give recruiters information about research being part of clinical care*, *the importance of maternity care research*, *rationale for trial*	**√**	**√**	**-**	**√**	**√**	**√**	Yes	Well received in workshop
5.3	**Information about social and environmental consequences**								
	Description: *Remind recruiters that all eligible women are entitled to be asked to participate in the trial*, *and by inviting all eligible women*, *it means the sample is more likely to be representative*, *maybe greater chances of trial success*, *impact on research*	**√**	**√**	**-**	**√**	**√**	**√**	Yes	Well received at workshop
11.2	**Reduce negative emotions**								
	Description: *Overcome recruiters’ feelings of apprehension from getting the feeling or the ‘vibe’ that the woman may not be interested in participating in the trial by reminding them it’s OK just to invite women by giving them information*	**√**	**√**	**-**	**√**	**√**	**√**	Yes	Well received at workshop
13.2	**Reframing**								
	Description: *Educating recruiters that intervention is about ‘inviting’ (offering the chance) and not necessary recruiting all eligible women. Offering the opportunity to take part in research in the way that health advice/information is offered to everybody*	**√**	**√**	**-**	**√**	**√**	**√**	Yes	Important concept to the aim of this intervention
5.6	**Information about emotional consequences**								
	Description: *Invite recruiters to share anecdotes of past emotionally sensitive situations where women were glad to be invited to participate in the trial and may have found inclusion helpful or comforting in their situation*	**√**	**√**	**-**	**√**	**√**	**√**	Yes	Well received at workshop
5.2	**Salience of consequences**								
	Description: *Invite previous trial invitees to share their experiences of being approached to take part in a trial in maternity care (*via* narrative testimonial*, *short video clip)*	**√**	**-**	**-**	**√**	**√**	**√**	No	Well received at workshop. Exclude as not target behaviour and difficult to ethically justify in APEASE

**Fig. 2:**
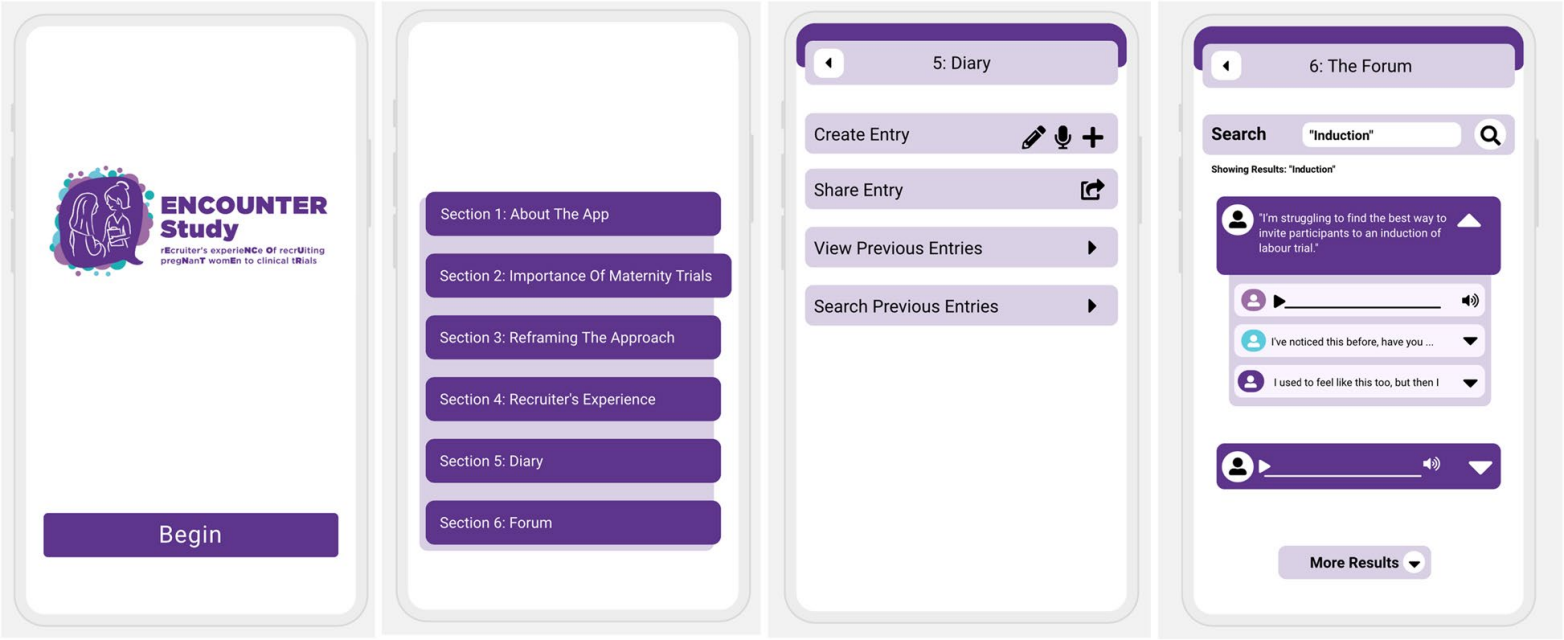
ENCOUNTER app prototype screenshots

Our choice of intervention delivery was informed by comments made by participants about the convenience and helpfulness of using mobile phone apps in their working practice and the high number of BCTs we wished to incorporate into one intervention. We developed a prototype app to deliver the intervention in this setting (Fig. 2). It should be noted, however, that in this study, we were interested in the BCT content of the intervention, rather than the technological functions of the app, and therefore, we focused predominantly on content during acceptability and feasibility assessment.

### Step 3—Evaluating acceptability and feasibility of the proposed intervention

The final step in the development process involved assessing the acceptability and feasibility of the intervention using an online survey capturing the opinions of key stakeholders. Fifty-four participants, twenty-five from Ireland and twenty-nine from the UK, initiated the online questionnaire, providing demographic information and answering the two statements questions. However, nine participants did not proceed beyond the two statement questions, resulting in a total of 45 completed questionnaires (reported in section below). Responses were provided by MHCPs, including midwives (clinical and research), research nurses, doctors (obstetricians and other specialties), allied health professionals, and maternity trial team members. All respondents described themselves as white and were predominately female 92.6% (*n* = *50*). Research midwives represented the largest group of respondents (*n* = 25, 46%), followed by clinical midwives (*n* = 8, 15%) and trial team members (*n* = 8, 15%). Detailed participant characteristics are presented in Table 7.

**Table 7:** Recruitment intervention survey: participant characteristics

Total survey responses*—n* = 54 (Ireland*—n* = 25, UK*—n* = 29) Total surveys completed*—n* = 45 *n (%)*								
Role	Clinical midwife	Research midwife	Clinical nurse	Research nurse	Doctor (obstetrics)	Doctor (other)	Allied health professional	Trial team member
Survey responses initiated	8	25	0	1	6	3	3	8
Dropped out before TFA questions	1	3	-	0	2	1	0	2
Completed TFA questions	7	22	-	1	4	2	3	6
*Based in*								
Ireland	7 (71.43)	5 (20)	-	1	2 (33.33)	3 (100)	3 (100)	4 (50)
UK	1 (28.57)	20 (80)	-	0	4 (66.67)	0	0	4 (50)
*Trial experience*								
< 2 years	4 (50)	4 (16)	-	0	1 (16.67)	0	0	2 (25)
2–5 years	0	9 (36)	-	0	2 (33.33)	2 (66.67)	0	0
> 5 years	4 (50)	12 (48)	-	1 (100)	3 (50)	1 (33.33)	3 (100)	6 (75)
*Age*								
18–24	0	0	-	0	0	0	0	0
25–34	0	4 (16)	-	0	2 (33.33)	2 (66.67)	2 (66.67)	3 (37.5)
35–44	3 (37.5)	8 (32)	-	0	0	1 (33.33)	0	1 (12.5)
45–54	4 (50)	10 (40)	-	1	2 (33.33)	0	1 (33.33)	3 (37.5)
55–64	1 (12.5)	3 (12)	-	0	2 (33.33)	0	0	1 (12.5)
65 +	0	0	-	0	0	0	0	0
*Ethnicity*								
White	8 (100)	25 (100)	-	1 (100)	6 (100)	3 (100)	3 (100)	8 (100)
*Gender*								
Male	0	1 (4)	-	0	1 (16.67)	0	1 (33.33)	2 (25)
Female	8 (100)	24 (96)	-	1 (100)	6 (83.33)	3 (100)	2 (66.67)	6 (75)
Other	0	0	-	0	0	0	0	0
Response to statement questions								
* “Improving recruitment to trials in maternity care is something I care about”*								
Strongly agree	37.5% (3)	80 (20)	-	0	83.33% (5)	50% (2)	100% (3)	100% (8)
Agree	62.5% (5)	20 (5)	-	0	16.67% (1)	50% (2)	0	0
Neutral	0	0	-	100%	0	0	0	0
Disagree	0	0	-	0	0	0	0	0
Strongly disagree	0	0	-	0	0	0	0	0
* “I have a role to play in helping to ensure all eligible people are invited to participate in a clinical trial in maternity care”*								
Strongly agree	0	76 (19)	-	0	66.67% (4)	50% (2)	66.67% (2)	100% (8)
Agree	100% (8)	24 (6)	-	100%	33.33% (2)	50% (2)	33.33% (1)	0
Neutral	0	0	-	0	0	0	0	0
Disagree	0	0	-	0	0	0	0	0
Strongly disagree	0	0	-	0	0	0	0	0

In response to the first statement question on the survey: “Improving recruitment to trials in maternity care is something I care about”, over 98% (*n* = *53*) of respondents agreed or strongly agreed. In response to the second statement: “I have a role to play in helping to ensure all eligible people are invited to participate in a clinical trial in maternity care”, 100% (*n* = *54*) agreed or strongly agreed with the statement.

Forty-five respondents proceeded to complete the TFA questions on the questionnaire. Below, we give a brief report on the responses to the seven TFA questions and provide detailed survey results in Table 8. Just over half (60%, *n* = 27) of respondents perceived that the ENCOUNTER app would be effective in helping MHCPs offer the opportunity to take part in a trial (*TFA construct: Perceived effectiveness*). More than 66% (*n* = *30*) of respondents were enthusiastic about using or seeing the app in use (*TFA construct: Affective attitude*). With regard to the burden of engaging with the app, over 77% (*n* = *35*) thought it would be easy or required some effort, however, 18% (*n* = *8*) believed it would be difficult or require a lot of effort to engage with it (*TFA construct: Burden*). Respondents were less positive when considering how using the app might impact other work practices, with 58% (*n* = 25) believing it would interfere with other tasks MHCPs need to do (*TFA construct: Anticipated opportunity*). Just over half (53%, *n* = 24) were confident or very confident about MHCPs being able to engage and use the app, while 47% *(n* = 21) of respondents were doubtful that MHCPs would be able to do so (*TFA construct: Self-efficacy*). When asked about the clarity of the proposed intervention, how it would be used, and how the app might enable more eligible people to be invited to trials, 73% (*n* = *33*) felt clear or somewhat clear in their understanding of how the intervention might work (*TFA construct: Intervention coherence*). When asked if they had any ethical concerns about the app, and how or if it would fit into their healthcare systems, 80% (*n* = *36*) of respondents had no ethical concerns. However, 53% (*n* = *24*) believed that the app would need to undergo some adjustments in order to fit in with the local health system (*TFA construct: Ethicality*).

**Table 8:** TFA online survey results: acceptability of the prototype intervention

		**Summative results**	**Clinical midwife**	**Research midwife**	**Research nurse**	**Doctor (obstetrics)**	**Doctor (other specialty)**	**Allied Health Professional**	**Trial Team member**
**TFA**	**Total number of responses across participant groups**	45	7	22	1	4	2	3	6
**Perceived effectiveness**	**Question 1***—How likely is it that the ENCOUNTER app will help Healthcare Professionals to recruit to maternity care trials? (i.e. Do you think more people will be offered the opportunity to take part in a trial?)*								
	Extremely likely	(2)	14% (1)	5% (1)	0	0	0	0	0
	likely	(25)	71% (5)	60% (13)	100% (1)	50% (2)	50% (1)	0	50% (3)
	No opinion	(10)	0	18% (4)	0	25% (1)	50% (1)	33% (1)	50% (3)
	Unlikely	(8)	14% (1)	18% (4)	0	25% (1)	0	67% (2)	0
	Extremely unlikely	-	0	0	0	0	0	0	0
**Affective attitude**	**Question 2***—As a healthcare professional*, *how do you feel about using the ENCOUNTER app? or As a member of the Trial Team*, *how do you feel about the ENCOUNTER app being used in your trial?*								
	Very enthusiastic	(3)	0	14% (3)	0	0	0	0	0
	enthusiastic	(27)	71% (5)	64% (14)	0	50% (2)	100% (2)	0	67% (4)
	No opinion	(7)	14% (1)	5% (1)	100% (1)	25% (1)	0	33% (1)	33% (2)
	Unenthusiastic	(8)	14% (1)	18% (4)	0	25% (1)	0	67% (2)	0
	Extremely enthusiastic	-	0	0	0	0	0	0	0
**Burden**	**Question 3***—How much effort would be required to engage with the ENCOUNTER app? or As a member of the Trial Team*, *how much effort would be required for the ENCOUNTER app being used in your trial?*								
	It’s easy, no effort required at all	(4)	14% (1)	9% (2)	0	0	0	0	17% (1)
	Some effort required	(31)	71.43% (5)	73% (16)	0	75% (3)	100% (2)	67% (2)	50% (3)
	No opinion	(2)	0	0	100% (1)	0	0	0	17% (1)
	A lot of effort is required	(7)	0	18% (4)	0	25% (1)	0	33% (1)	17% (1)
	It’s difficult, a great deal of effort is required	(1)	14.29% (1)	0	0	0	0	0	0
**Anticipated opportunity**	**Question 4***—Do you think using the ENCOUNTER app would interfere with other tasks Healthcare Professionals need to do?*								
	No, using the app would easily fit in with other tasks	(3)	14% (1)	5% (1)	0	0	0	0	17% (1)
	Using the app would not interfere with other tasks	(16)	29% (2)	36% (8)	0	75% (3)	0	67% (2)	17% (1)
	No opinion	(11)	29% (2)	27% (6)	100% (1)	0	50% (1)	0	17% (1)
	Using the app would interfere with other tasks	(15)	29% (2)	32% (7)	0	25% (1)	50% (1)	33% (1)	50% (3)
	Yes, using the app would be disruptive to other tasks	-	0	0	0	0	0	0	0
**Self-efficacy**	**Question 5***—How confident are you that Healthcare Professionals will be able to engage with and use the ENCOUNTER app?*								
	Very confident	(2)	0	5% (1)	0	0	0	33% (1)	0
	Confident	(22)	57% (4)	55% (12)	0	75% (3)	50% (1)	0	33% (2)
	No opinion	(10)	14% (1)	18% (4)	100% (1)	0	50% (1)	0	50% (3)
	Doubtful	(11)	29% (2)	23% (5)	0	25% (1)	0	67% (2)	17% (1)
	Very doubtful	-	0	0	0	0	0	0	0
**Intervention coherence**	**Question 6***—*Is it clear to you how the ENCOUNTER app would be used and how it might enable all eligible people to participate in a clinical trial in maternity care?								
	Yes, it’s very clear	(11)	43% (3)	27% (6)	0	25% (1)	50% (1)	0	0
	It’s somewhat clear	(22)	43% (3)	55% (12)	0	50% (2)	50% (1)	33% (1)	50% (3)
	No opinion	(4)	0	0	100% (1)	25% (1)	0	33% (1)	17% (1)
	It’s not clear	(7)	14% (1)	13.64% (3)	0	0	0	33% (1)	33% (2)
	No, it’s very unclear	(1)	0	5% (1)	0	0	0	0	0
**Ethicality**	**Question 7***—Do you have any ethical concerns about the ENCOUNTER app being used? (i.e. do you think the ENCOUNTER App is a good fit with the existing healthcare system)*								
	No ethical concerns, the app would fit very well with the healthcare system	(12)	29% (2)	23% (5)	0	50% (2)	50% (1)	0	33% (2)
	No ethical concerns, but app may need adjustments to fit with the healthcare system	(24)	71% (5)	59% (13)	0	25% (1)	50% (1)	100% (3)	17% (1)
	No opinion	(5)	0	14% (3)	100% (1)	0	0	0	17% (1)
	Some ethical concerns, would need adjustment to fit with the healthcare system	(4)	0	5% (1)	0	25% (1)	0	0	33% (2)
	I have ethical concerns, the app would not be a good fit with the healthcare system	-	0	0	0	0	0	0	0

*NB* No clinical nurses took part in the survey

## Discussion

The majority of existing recruitment interventions concentrate on modifying the information given to potential participants about the trials [3], and do not address recruitment staff training, despite being identified as a top priority for recruitment intervention development [32]. Townsend et al.’s systematic review of trial recruiter training programmes found most evidence originated within the oncology setting, where interventions tended to be delivered as multidisciplinary workshops covering generic and trial specific issues [33]. The review authors note that while there is limited evidence to support the efficacy of any particular training programme, some interventions were reported to increase HCPs communication skills. However, as this increase was self-reported, with no indication of the duration over which the change was sustained, caution should be observed when interpreting this finding. While there is an absence of recruiter training interventions focused on maternity trial recruitment, there has been recent research activity in non-trial specific maternity recruitment research [34, 35]. A qualitative exploration of midwives recruiting to maternity research in a hospital setting found the competing worlds of clinical practice and clinical research governed midwives motivation to recruit [34]. The authors recommended involving clinicians in planning and designing strategies to overcome factors that inhibit recruitment [34]. Rose et al.’s study, a theory-informed online survey which included 22 community midwives recruiting to maternity research studies, identified six TDF domains as key influencers in the perceived barriers and enablers to approaching patients about research participation [35]. Four domains (*Environmental context and resources*, *Knowledge*, *Social Influences*, *Goals*) share commonality with the eight salient domains identified in our previous findings [22]. However, the domain *Behavioural Regulatio*n was reported as rarely evident by Rose et al., which is contrary to our findings, where *Behavioural Regulation* featured as a salient domain more than once. While the authors suggest addressing behavioural barriers and enabling midwives to approach potential participants requires a package of theory-based interventions, they stop short of specifying which BCTs to include in the package [35].

The opportunities for behavioural theory to improve clinical trials is being recognised across several trial processes [17]. Recent examples in the literature show behavioural approaches being used as a ‘diagnostic’ tool to identify recruitment problems [15, 19, 36]. The advantages of adopting this approach are demonstrated by Brehaut et al. in their study with HCPs recruiting to a trial concerning recurrent venous thromboembolism [15], and Castillo et al. in their study concerning haematologists recruiting patients to a CAR(T) cell therapy trial [19], where the use of behavioural theory revealed a variety of additional factors impeding recruiters that had not been uncovered by previous research efforts. Previous studies have largely focused on ‘diagnosing’ recruitment problems; however, behavioural theory can move beyond ‘diagnostics’ towards ‘treating’ recruitment problems by informing the development of behaviour change interventions [21, 37]. Ellis et al. take this approach in their study developing an implementation intervention for cancer clinical trials aimed at HCPs [21]. The authors ‘brainstormed’ BCTs and potential modes of delivery, to iteratively shape a multi-modal trial process intervention to align with opportunities and support structures within the context of community practice. Of the 27 different BCTs identified by Ellis et al., six shared commonality with our intervention: *Information about social and environmental consequences*, *framing/reframing*, *review outcome goals*, *goal setting*, *self-monitoring*, and *information about consequence*s. However, there were several task performance orientated BCTs (i.e. instructions, demonstrations, how to perform) that were not identified in our work. Ellis et al. used a mixture of in-person, telephone, and electronic communications and, similar to our approach, chose online video as a supportive mode of delivery for the intervention in a practice setting [21]. Other teams have explored the use of behavioural theory to develop patient-centred interventions to improve trial participant retention [37]. Newlands et al.’s. study explored two interventions, selected by participants, that centred on goal setting and motivational information. Their goal setting intervention was based on a single BCT (1.1*—Goal setting behaviour*) for communication from the HCP point of contact or peers from trial. Their motivational information intervention included three BCTs (*5.1—Information about health consequences*; *5.2—Salience of consequences*; *5.3—Information about social and environmental consequences*) for communication between participant and trial office at various key touch points in the trial. The intended mode of delivery for both interventions was by verbal, paper based, or electronic means. While the above BCTs also featured in our intervention, the mode of delivery differed. Our intervention included a large number of BCTs and made innovative use of the feedback on BCTs to guide the mode of delivery for the intervention.

There are few examples of trial recruitment intervention development in the literature that use stakeholder accounts or make explicit reference to in-depth assessment of the HCP reported recruitment challenges. The QuinteT Recruitment Intervention (QRI) is an example where stakeholder experiences have been incorporated into the interventions and successfully improved recruitment rates [38]. The QRI is a two-phase intervention that gathers evidence at the clinical site(s) about trial specific recruitment processes and then produces a bespoke plan to address the difficulties [38]. We utilised stakeholder involvement in a different way. From inception onwards, we included MHCP recruiters through all stages of development, building on the accounts of MHCP recruiters from a variety of trials in maternity care. In addition, we included maternity service users in the co-design phase and maternity trial teams in the acceptability and feasibility stage. This stakeholder responsive approach allowed us to include additional BCTs and tailor the prototype, to produce a proactive pragmatic intervention primed for success from the outset.

Evaluation is key for all interventions. It is essential that theory-informed trial recruitment interventions are evaluated in a robust manor to determine whether or not the evidence from changing health behaviours extends to changing MHCPs trial behaviours [37]. One method to consider for evaluation is a Study Within A Trial (SWAT). The self-contained research study embedded within a host trial, offers the potential to test the intervention in a real-world context while making a contribution to the pool of methodological evidence [14]. We kept the necessity for evaluation foremost during development. To pave the way for future robust evaluation, we ensured our intervention aligned with the NIHR Framework for the development and evaluation of complex interventions [13].

### Strengths/limitations

We developed a theoretically grounded intervention that was acceptable to MHCPs and maternity trial teams, across Ireland and the UK. While our results are limited to these countries, they may be transferable to regions beyond, but it would be important to determine whether the behavioural barriers present in other countries were similar to those identified in our initial analysis. A key strength of our study was stakeholder engagement. We included MHCP recruiters, maternity service users, and maternity trial team members, at key stages of intervention design and development. Perspectives from MHCPs with experience across multiple maternity trials, covering a range of clinical areas and interventions in maternity care, fed directly into the development of the intervention, increasing the transferability of our findings. Holding two separate online workshops strengthened our findings as it allowed time for rich discussion and helped stave off potential power imbalances by achieving an even spread of participants from different clinical roles across workshops. However, we acknowledge the influence of potential bias in the workshop allocation in relation to the different agendas planned for each workshop. There were limitations in the diversity of our stakeholder groups, and the sample is not a direct reflection of the population the intervention was developed for. MHCPs in this study had previously participated in our qualitative interview study [22] and may have been more agreeable to the proposed potential solution. In mitigation of this, we assessed the acceptability of the intervention using an online survey, to potentially reach a larger more diverse group of stakeholders. However, despite our efforts, the sample demographics of the survey revealed a lack of heterogeneity.

In using behavioural theory, despite the guidance of empirically derived tools, we acknowledge that there remains a degree of subjectivity. The application of APEASE criteria is not an exact science and other researchers may have categorised differently. However, applying APEASE criteria is also a strength as it ensures the content of an intervention is tailored to the specific context. Using behavioural theory and co-design in tandem to approach intervention development added synergistic value beyond that to be gained by using either approach independently [39].

## Conclusion

This study reports the development of a theory-based, codesigned intervention aimed at changing the behaviour of MHCPs tasked with approaching all eligible potential participants and inviting then to join a trial in maternity care. We used findings from our previous interview study with MHCP recruiters [22] and applied theory and co-design principles to develop each component of the intervention. We welcome rigorous evaluation of our prototype in a SWAT, primarily to establish if the intervention enables MHCPs to invite all eligible people to participate in a maternity care trial and secondly, to determine if this had an impact on the overall rate of recruitment to the trial. While recruitment to trials in maternity care is highly complex and not limited to changing the behaviour of MHCPs alone, we are hopeful that the theoretical grounding of our intervention will support healthcare professionals to invite all eligible people to participate in clinical trial in maternity care.

## Supplementary Information

**Additional file 1: Supp. File 1:** GUIDED checklist and TIDieR checklist.

## Data Availability

The full set of data supporting the conclusions of this article are available as additional files. Individual participant data will not be shared. However, we invite queries and comments from interested readers regarding the data or analytic process.

## References

[1] Walters SJ, Bortolami O, Flight L, Hind D, Jacques RM, Bonacho Dos AnjosHenriques-Cadby I (2017). Recruitment and retention of participants in randomised controlled trials: a review of trials funded and published by the United Kingdom Health Technology Assessment Programme. BMJ Open..

[2] Button KS, Ioannidis JP, Mokrysz C, Nosek BA, Flint J, Robinson ES (2013). Power failure: why small sample size undermines the reliability of neuroscience. Nat Rev Neurosci.

[3] Treweek S, Pitkethly M, Cook J, Fraser C, Mitchell E, Sullivan F (2018). Strategies to improve recruitment to randomised trials. Cochrane Database Syst Rev..

[4] Salman RA-S, Beller E, Kagan J, Hemminki E, Phillips RS, Savulescu J (2014). Increasing value and reducing waste in biomedical research regulation and management. Lancet..

[5] van der Zande ISE, van der Graaf R, Hooft L, van Delden JJM (2018). Facilitators and barriers to pregnant women’s participation in research: a systematic review. Women Birth.

[6] Ballantyne A, Rogers W. Pregnancy, vulnerability, and the risk of exploitation in clinical research. In: Baylis FB, A., editor. Clinical Research Involving Pregnant Women Research Ethics Forum. 3. Switzerland: Springer; 2016. 10.1007/978-3-319-26512-4. ISBN978-3-319-26512-4.

[7] Hanrahan V, Gillies K, Biesty L (2020). Recruiters’ perspectives of recruiting women during pregnancy and childbirth to clinical trials: a qualitative evidence synthesis. PLoS ONE.

[8] Heyrana K, Byers HM, Stratton P (2018). Increasing the participation of pregnant women in clinical trials. JAMA.

[9] Tooher RL, Middleton PF, Crowther CA (2008). A thematic analysis of factors influencing recruitment to maternal and perinatal trials. BMC Pregnancy Childbirth.

[10] Monaghan H, Richens A, Colman S, Currie R, Girgis S, Jayne K (2007). A randomised trial of the effects of an additional communication strategy on recruitment into a large-scale, multi-centre trial. Contemp Clin Trials.

[11] Tilley B, Mainous A, Elm J, Pickelsimer E, Soderstrom L, Ford M (2012). A randomized recruitment intervention trial in Parkinson’s disease to increase participant diversity- early stopping for lack of efficacy. Clin Trials.

[12] Treweek S, Barnett K, Maclennan G, Bonetti D, Eccles MP, Francis JJ (2012). E-mail invitations to general practitioners were as effective as postal invitations and were more efficient. J Clin Epidemiol.

[13] Skivington K, Matthews L, Simpson SA, Craig P, Baird J, Blazeby JM (2021). Framework for the development and evaluation of complex interventions: gap analysis, workshop and consultation-informed update. Health Technol Assess.

[14] Treweek S, Bevan S, Bower P, Campbell M, Christie J, Clarke M (2018). Trial Forge Guidance 1: what is a Study Within A Trial (SWAT)?. Trials.

[15] Brehaut JC, Lavin Venegas C, Hudek N, Presseau J, Carroll K, Rodger M (2021). Using behavioral theory and shared decision-making to understand clinical trial recruitment: interviews with trial recruiters. Trials.

[16] Delaney H, Devane D, Hunter A, Treweek S, Mills N, Gamble C, Smith V. A concept analysis of 'trial recruitment' using the hybrid model. HRB Open Research. 2022;3:92. 10.12688/hrbopenres.13173.2. eCollection 2020. 10.12688/hrbopenres.13173.1 PMC9021666 35510227

[17] Gillies K, Brehaut J, Coffey T, Duncan EM, Francis JJ, Hey SP (2021). How can behavioural science help us design better trials?. Trials.

[18] Michie S, Atkins L, West R (2014). The behaviour chane wheel: a guide to designing interventions. 1 ed.

[19] Castillo G, Lalu M, Asad S, Foster M, Kekre N, Fergusson D (2021). Hematologists’ barriers and enablers to screening and recruiting patients to a chimeric antigen receptor (CAR) T cell therapy trial: a theory-informed interview study. Trials.

[20] Guillot M, Asad S, Lalu MM, Lemyre B, Castillo G, Thebaud B (2019). So you want to give stem cells to babies? Neonatologists and parents’ views to optimize clinical trials. J Pediatr.

[21] Ellis S, Geana M, Griebling T, McWilliams C, Gills J, Stratton K (2019). Development, acceptability, appropriateness and appeal of a cancer clinical trials implementation intervention for rural- and minority-serving urology practices. Trials.

[22] Hanrahan V, Biesty L, Lawrie L, Duncan E, Gillies K. Theory-guided interviews identified behavioral barriers and enablers to healthcare professionals recruiting participants to maternity trials. J Clin Epidemiol. 2022;145:81–91. 10.1016/j.jclinepi.2022.01.015. Epub 2022 Jan 23. 10.1016/j.jclinepi.2022.01.015 35081447

[23] Presseau J, McCleary N, Lorencatto F, Patey AM, Grimshaw JM, Francis JJ (2019). Action, actor, context, target, time (AACTT): a framework for specifying behaviour. Implement Sci.

[24] Michie S, Richardson M, Johnston M, Abraham C, Francis J, Hardeman W (2013). The behavior change technique taxonomy (v1) of 93 hierarchically clustered techniques: building an international consensus for the reporting of behavior change interventions. Ann Behav Med.

[25] Carey RN, Connell LE, Johnston M, Rothman AJ, de Bruin M, Kelly MP (2019). Behavior change techniques and their mechanisms of action: a synthesis of links described in published intervention literature. Ann Behav Med.

[26] Michie SAL, West R (2014). The APEASE criteria for designing and evaluating interventions. In: The Behaviour Change Wheel: A Guide to Designing Interventions.

[27] Farr M (2018). Power dynamics and collaborative mechanisms in co-production and co-design processes. Crit Soc Policy.

[28] Hsieh HF, Shannon SE (2005). Three approaches to qualitative content analysis. Qual Health Res.

[29] Duncan E, O’Cathain A, Rousseau N, Croot L, Sworn K, Turner KM (2020). Guidance for reporting intervention development studies in health research (GUIDED): an evidence-based consensus study. BMJ Open.

[30] Hoffmann TC, Glasziou PP, Boutron I, Milne R, Perera R, Moher D (2014). Better reporting of interventions: template for intervention description and replication (TIDieR) checklist and guide. BMJ.

[31] Sekhon M, Cartwright M, Francis JJ (2017). Acceptability of healthcare interventions: an overview of reviews and development of a theoretical framework. BMC Health Serv Res.

[32] Bower P, Brueton V, Gamble C, Treweek S, Smith CT, Young B, Williamson P. Interventions to improve recruitment and retention in clinical trials: a survey and workshop to assess current practice and future priorities. Trials. 2014;15:399. 10.1186/1745-6215-15-399. 10.1186/1745-6215-15-399 PMC4210542 25322807

[33] Townsend D, Mills N, Savovic J, Donovan JL (2015). A systematic review of training programmes for recruiters to randomised controlled trials. Trials.

[34] Daly D, Hannon S, Brady V (2019). Motivators and challenges to research recruitment - a qualitative study with midwives. Midwifery.

[35] Rose J, Lynn K, Akister J, Maxton F, Redsell SA (2021). Community midwives’ and health visitors’ experiences of research recruitment: a qualitative exploration using the Theoretical Domains Framework. Prim Health Care Res Dev.

[36] Ellis SD, Geana M, Mackay CB, Moon DJ, Gills J, Zganjar A (2019). Science in the Heartland: exploring determinants of offering cancer clinical trials in rural-serving community urology practices. Urol Oncol..

[37] Newlands R, Duncan E, Treweek S, et al. The development of theory-informed participant-centred interventions to maximise participant retention in randomised controlled trials. Trials. 2022;23:268. 10.1186/s13063-022-06218-8. 10.1186/s13063-022-06218-8 PMC8994320 35395930

[38] Donovan J, Rooshenas L, Jepson M, Elliott D, Wade J, Avery K (2016). Optimising recruitment and informed consent in randomised controlled trials: the development and implementation of the Quintet Recruitment Intervention (QRI). Trials.

[39] O’Cathain A, Croot L, Sworn K, Duncan E, Rousseau N, Turner K (2019). Taxonomy of approaches to developing interventions to improve health: a systematic methods overview. Pilot Feasibility Stud.

